# Acute thrombus formation on phosphorilcholine surface modified flow diverters

**DOI:** 10.1136/neurintsurg-2017-013175

**Published:** 2017-07-08

**Authors:** Miklos Marosfoi, Frederic Clarencon, Erin T Langan, Robert M King, Olivia W Brooks, Takamisu Tamura, John M Wainwright, Matthew J Gounis, Srinivasan Vedantham, Ajit S Puri

**Affiliations:** 1 New England Center for Stroke Research, Department of Radiology, University of Massachusetts Medical School, Worcester, Massachusetts, USA; 2 Department of Interventional Neuroradiology, Pitié-Salpêtrière Hospital, Paris, France; 3 Research and Development, Medtronic Neurovascular, Irvine, California, USA; 4 Department of Medical Imaging, The University of Arizona – Banner University Medical Center, Tucson, Arizona, USA

**Keywords:** optical coherence tomography, flow diverters, intracranial aneurysms, dual antiplatelet therapy

## Abstract

**Purpose:**

Thromboembolic complications remain a limitation of flow diverting stents. We hypothesize that phosphorilcholine surface modified flow diverters (Pipeline Flex with Shield Technology, sPED) would have less acute thrombus formation on the device surface compared with the classic Pipeline Embolization device (cPED).

**Methods:**

Elastase-induced aneurysms were created in 40 rabbits and randomly assigned to receive cPED or sPED devices with and without dual antiplatelet therapy (DAPT) (four groups, n=10/group). Angioplasty was performed to enhance apposition and create intimal injury for a pro-thrombotic environment. Both before and after angioplasty, the flow diverter was imaged with intravascular optical coherence tomography. The outcome measure was the number of predefined segments along the implant relative to the location of the aneurysm with a minimum of 0 (no clot formation) and maximum of 3 (all segments with thrombus). Clot formation over the device at ostia of branch arteries was assessed as either present or absent.

**Results:**

Following angioplasty, the number of flow diverter segments with clots was significantly associated with the flow diverter (p<0.0001), but not with DAPT (p=0.3872) or aneurysm neck size (p=0.8555). The incidence rate for clots with cPED was 1.72 times more than with sPED. The clots on the flow diverter at the location corresponding to side branch ostia was significantly lower with sPED than with cPED (OR 0.180; 95% CI 0.044 to 0.734; p=0.0168), but was not associated with DAPT (p=0.3198).

**Conclusion:**

In the rabbit model, phosphorilcholine surface modified flow diverters are associated with less thrombus formation on the surface of the device.

## Introduction

In the past 10 years, flow diverters (FDs) have been successfully used to treat large and giant unruptured intracranial aneurysms, primarily located in the anterior circulation.^1 2^ A recently published study demonstrated a high and gradually increasing aneurysm occlusion rate (95.2%) over a 5-year follow-up period^3^; however, in multiple large studies, major ipsilateral ischemic strokes have been reported with a range between 1.6% and 4.3%.^1 2^ Prevention of thromboembolic events is complicated by variable response to antiplatelet therapy, unpredictable patient compliance with medication, potential drug interactions, and the presence of comorbidities. These factors may all contribute to the formation of thrombi on the surface of the FDs during the procedure, which eventually serves as a source of emboli.^4^ Depending on the eloquence of affected brain parenchyma, the patient may suffer significant neurological deficits. Although the risk of ischemic events gradually decreases over time after device implant, it may be present until the endothelialization is completed and the FD is fully incorporated into the remodeled artery.

In an effort to decrease the periprocedural thromboembolic complications, phosphorilcholine (PC) coated stents were developed and assessed in cardiology.^5^ However, this experience cannot be fully translated to neuroendovascular procedures due to differences in vessel size (parent vessel and side branches), hemodynamic environment, and overall device engineering.

**Figure 1:**
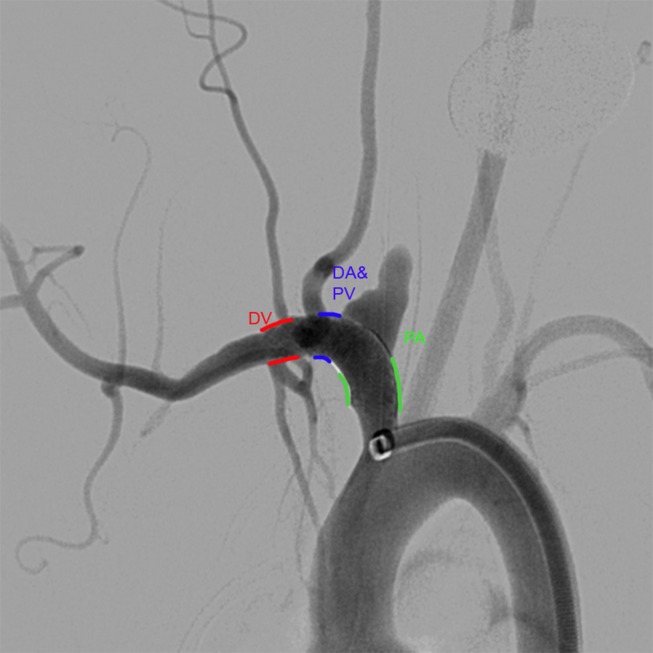
Anatomical locations for scoring thrombus formation. Summation of the number of locations with clot appreciated on the surface of the device resulting in scores ranging from 0 to 3. DV, distal to vertebral artery; DA&PV, distal to the aneurysm and proximal to the vertebral artery; PA, proximal to aneurysm.

Recently, a surface modified FD that covalently bonds 3 nm of PC to the braid wires has been introduced (Pipeline Flex with Shield Technology, Medtronic Neurovascular, Irvine, California, USA).^6^ We hypothesized that the Pipeline Flex with Shield Technology (sPED) would have less thrombus formation on the surface of the device compared with the classic Pipeline device (cPED). Furthermore, we assessed the consequences of angioplasty (after FD implant) on acute clot formation and evaluated how successfully this unwanted complication could be eliminated by using the Shield device. Our outcome measure was clot formation observed in vivo using optical coherence tomography (OCT) at three locations along the surface of the FD (figure 1). Clot formation was assessed as present (1) or absent (0) at each of these locations and summed for each animal, giving a maximum score of 3 and a minimum score of 0. Additionally, clot formation at the origin of covered side branch arteries was scored as a binary output (present or absent).

## Materials and methods

### Aneurysm creation and study design

After Institutional Animal Care and Use Committee approval, elastase-induced aneurysms were created in 40 New Zealand white rabbits at the origin of the right common carotid artery as previously described.^7^ A minimum of 3 weeks after creation, the aneurysms were screened via non-invasive intra-arterial digital subtraction angiography (DSA)^8^ and randomized to one of the following treatment groups: (1) cPED with animal on dual antiplatelet therapy (DAPT); (2) sPED with DAPT; (3) cPED without DAPT; and (4) sPED without DAPT. Animals assigned to DAPT received oral aspirin (10 mg/kg) and clopidogrel (10 mg/kg) beginning 5 days prior to FD implant and continued for the duration of the study.

### Flow diverter implant and OCT imaging

All procedures were performed under general anesthesia. First, the animals were pre-anesthetized by a subcuticular injection of atropine (0.01 mg/kg), followed by an intramuscular injection of ketamine (35 mg/kg) and xylazine (5 mg/kg) for anesthesia induction. Mechanical ventilation was maintained with 1–3%  isoflurane. Vitals were continuously monitored and respiration rate, oxygen saturation, end-tidal CO_2_, heart rate, and temperature were recorded.

The right common femoral artery was surgically exposed and a 6Fr introducer sheath was inserted. A Navien 072 was used to navigate the FD to the right subclavian artery. Both devices were sized based on the diameter of the brachiocephalic trunk proximal to the aneurysm and ranged from 3.75 to 4.5 mm. All devices were 16 mm in length. The device was deployed under fluoroscopic guidance and implanted once complete coverage of the aneurysm neck was documented. Angioplasty was performed on all devices (HyperGlide 4 mm x 10 mm) to optimize apposition and create a more aggressive environment for clot formation.

Before and after angioplasty, OCT imaging (Dragonfly Catheter, St Jude Medical, Westford, Massachusetts, USA) was performed in order to quantify the clot formation on the surface of the FD. The OCT images were acquired with a 10 mm/s pull-back speed through 54 mm length, generating 540 frames/pull-back. For sufficient blood clearance, iodinated contrast (Omnipaque 240, GE Healthcare, Malborough, Massachusetts, USA) was administered by a power injector at a flow rate of 4 mL/s for 4 s. In each case all 540 images acquired pre- and post-angioplasty were analyzed in a blinded fashion at three segments (distal to the vertebral artery (DV); distal to the aneurysm but proximal to the vertebral artery (DA&PV); and proximal to the aneurysm (PA); figure 1). The number of segments with clots (count variable) was the outcome variable of interest. Thus, there could be a maximum of three segments and a minimum of zero. In addition, clot formation at the aneurysm neck and at locations corresponding to side branches—namely the internal thoracic, vertebral, and cervical arteries—were quantified for presence/absence (binary variable). Aspirin reaction units (ARU) and P2Y12 reaction units (PRU) were measured prior to randomization to treatment group and at the time of implant with the VerifyNow instrument (Accriva, San Diego, California, USA). Aneurysm occlusion was assessed prior to euthanasia at 30 days on a previously described 4-point scale.^9^

### Statistical methods

For the outcome variable of interest (ie, number of segments with clots), Poisson regression that is appropriate for the count variable was used for modeling. The independent variables of interest were the FD (sPED vs cPED), DAPT (yes or no), and the aneurysm neck size (mm). A χ^2^ goodness-of-fit test was used to assess if the Poisson model form fit the data. In order to assess if clots formed at the aneurysm neck and at the location corresponding to side branches were associated with the independent variables, multivariable logistic regression models that were appropriate for binary data were used. All multivariable logistic regression models employed Firth’s bias correction and Hosmer–Lemeshow goodness-of-fit were assessed. For the cohort that underwent DAPT, the differences in ARU and PRU from time of implant to baseline were quantified. Shapiro–Wilk’s test was used to test for normality and either a paired t-test or Wilcoxon signed rank sum test was used to analyze the effectiveness of DAPT.

## Results

### Aneurysm and parent vessel characteristics

Randomization was effective, and there were no differences in baseline aneurysm characteristics (table 1). The aneurysm occlusion rates at 30 days across the four groups were not different (p=0.8512, Kruskal–Wallis test) and were independent of DAPT administration and device type. In each group at 30-day follow-up imaging, half of the aneurysms were either completely occluded or had a small neck remnant, and the remaining half continued to have filling of the aneurysm dome.

**Table 1:** Baseline characteristics of the aneurysm, parent vessel dimensions, platelet function, and the devices deployed

	cPED		sPED		p Value
	No DAPT	DAPT	No DAPT	DAPT	
Aneurysm neck size (mm)	4.1±1.3	3.8±0.7	3.9±1.0	4.3±1.8	0.78
Aneurysm width (mm)	3.8±0.9	3.3±0.8	3.8±0.9	3.4±1.0	0.51
Aneurysm height (mm)	7.7±2.2	6.3±1.2	7.6±2.5	7.7±2.3	0.33
Parent vessel diameter proximal to aneurysm (mm)	3.7±0.3	4.0±0.6	3.6±0.4	3.9±0.4	0.18
Parent vessel diameter distal to aneurysm (mm)	3.7±0.8	3.6±0.4	3.5±0.5	3.9±0.8	0.58
Subclavian artery diameter (mm)	2.2±0.2	2.3±0.3	2.2±0.3	2.4±0.4	0.30
PRU	261±48	56±26	249±52	84±26	<0.0001
ARU	661±6	642±43	636±57	657±12	0.90
Device diameter (mm)	4.0	4.0	4.0	4.125	0.58
Proximal landing zone					NS
Innominate artery	9	9	9	9	
Herniation into aorta	1	1	1	1	

All quantities expressed as mean±SD, except device diameters (median) and proximal landing zone (number of animals).

ARU, aspirin reaction units; cPED, classic Pipeline Embolization Device; DAPT, dual antiplatelet therapy; PRU, P2Y12 reaction units; sPED, Pipeline Flex with Shield Technology.

### Effectiveness of DAPT

For the cohort that received DAPT, the difference in PRU prior to and 5 days after DAPT was initiated did not satisfy the normality assumption (p=0.0014). The Wilcoxon signed rank sum test indicated that the PRU 5 days after DAPT was significantly lower than before administration of clopidogrel (median difference −164 (Q1, Q3 −170, –148), p=0.0039). At the time of implant, animals receiving DAPT had a significantly lower PRU (median 67 vs 251, respectively; p<0.0001, unpaired two-tailed t-test after satisfying normality test) than those not receiving antiplatelet medications. For the cohort that underwent DAPT, the difference in ARU before and 5 days after administering DAPT marginally satisfied the normality assumption (p=0.0810). The paired t-test indicated that the ARU was lower 5 days after DAPT than at baseline (mean±SD difference −6.833±11.72), but was not significant (p=0.2126). ARU values were not different between animals administered aspirin and those not given aspirin (p=0.75, unpaired two-tailed Mann–Whitney test).

### Number of FD segments with clots

**Figure 2:**
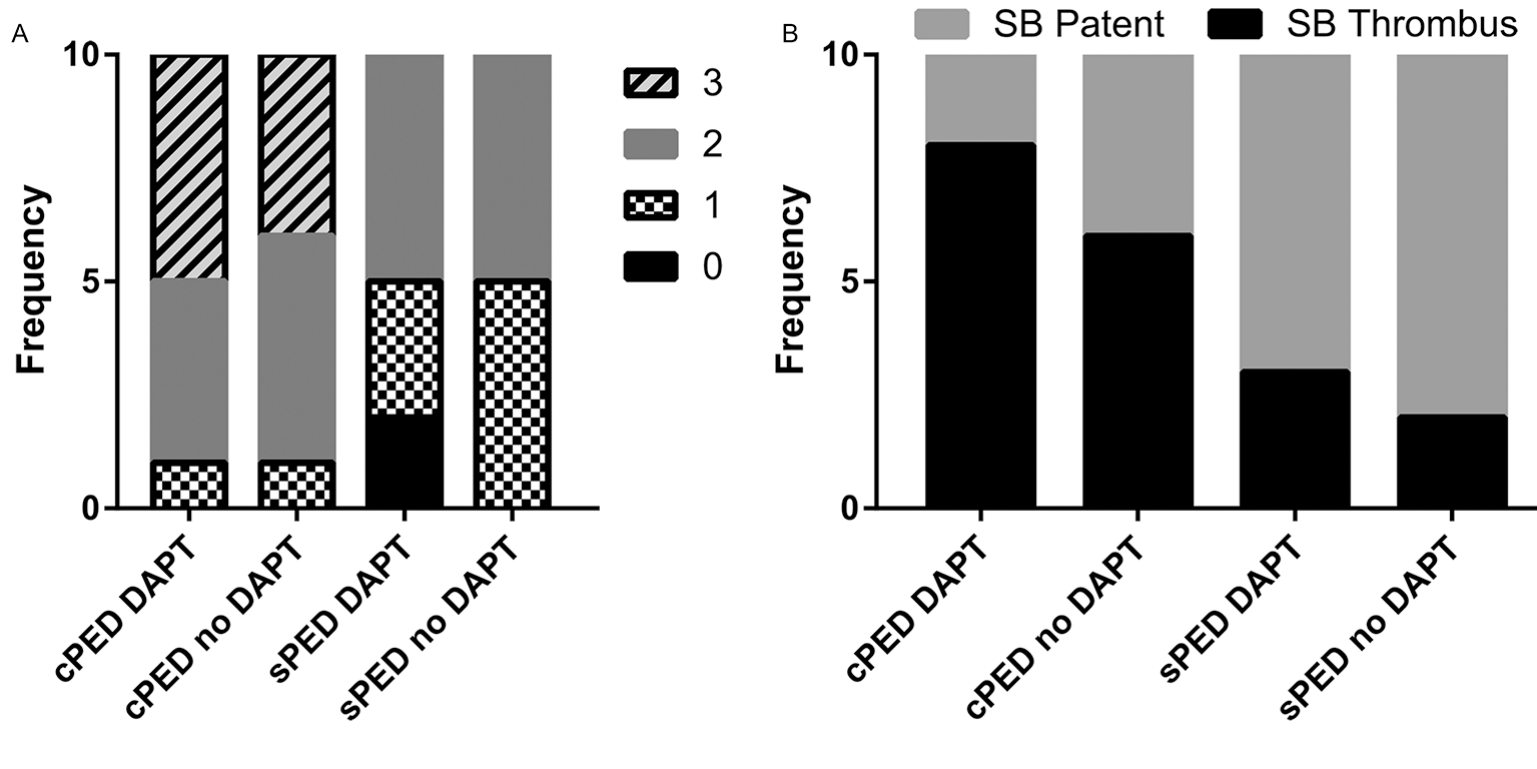
Frequency of clot scores in each group. (A) Immediately after angioplasty during the implant procedure there is a significant reduction in the number of locations with visible clot on optical coherence tomography (OCT) for the Shield Pipeline Embolization Device (sPED) and no appreciable difference as a function of dual antiplatelet treatment (DAPT). (B) Immediately after angioplasty following device implantation the frequency of clots forming along the surface of the device covering the ostia of side branch arteries (SB) is reduced with the new generation flow diverter (sPED) and did not change as a function of DAPT.

Prior to angioplasty, the number of FD segments with clots quantified using OCT were not associated with the FD (p=0.8279), DAPT (p=0.5177), or neck size (p=0.4363). χ^2^ goodness-of-fit showed that the Poisson model form fit the data (p=0.999). After angioplasty the number of FD segments with clots quantified using OCT (figure 2A) was significantly associated with the FD (p<0.0001), but not with DAPT (p=0.3872) or neck size (p=0.8555). When neck size and the use of DAPT are held constant, the incidence rate for clots with cPED was 1.72 times more than that of sPED. χ^2^ goodness-of-fit showed that the Poisson model form fit the data (p=0.998).

### Clots on FD at the location corresponding to side branches

Prior to angioplasty, the clots on the FD at the location corresponding to side branches were not associated with the FD (p=0.3169), DAPT (p=0.7009), or neck size (p=0.9115). Hosmer–Lemeshow goodness-of-fit was satisfied (p=0.1798). Post angioplasty, the clots on the FD at the location corresponding to side branches (figure 2B) were significantly lower with sPED than with cPED (OR 0.180; 95% CI 0.044 to 0.734; p=0.0168), but were not associated with DAPT (p=0.3198) or neck size (p=0.6610). Hosmer–Lemeshow goodness-of-fit was satisfied (p=0.3119).

### Clots on FD at the location corresponding to aneurysm neck

Prior to angioplasty, the clots on the FD at the location corresponding to the aneurysm neck were not associated with the FD (p=0.4399), DAPT (p=0.5537), or neck size (p=0.9651). Hosmer–Lemeshow goodness-of-fit was satisfied (p=0.9440). Post angioplasty, the clots on the FD at the location corresponding to the aneurysm neck were not associated with the FD (p=0.9374), DAPT (p=0.9263), or neck size (p=0.6546). Hosmer–Lemeshow goodness-of-fit was satisfied (p=0.7252).

## Discussion

**Figure 3:**
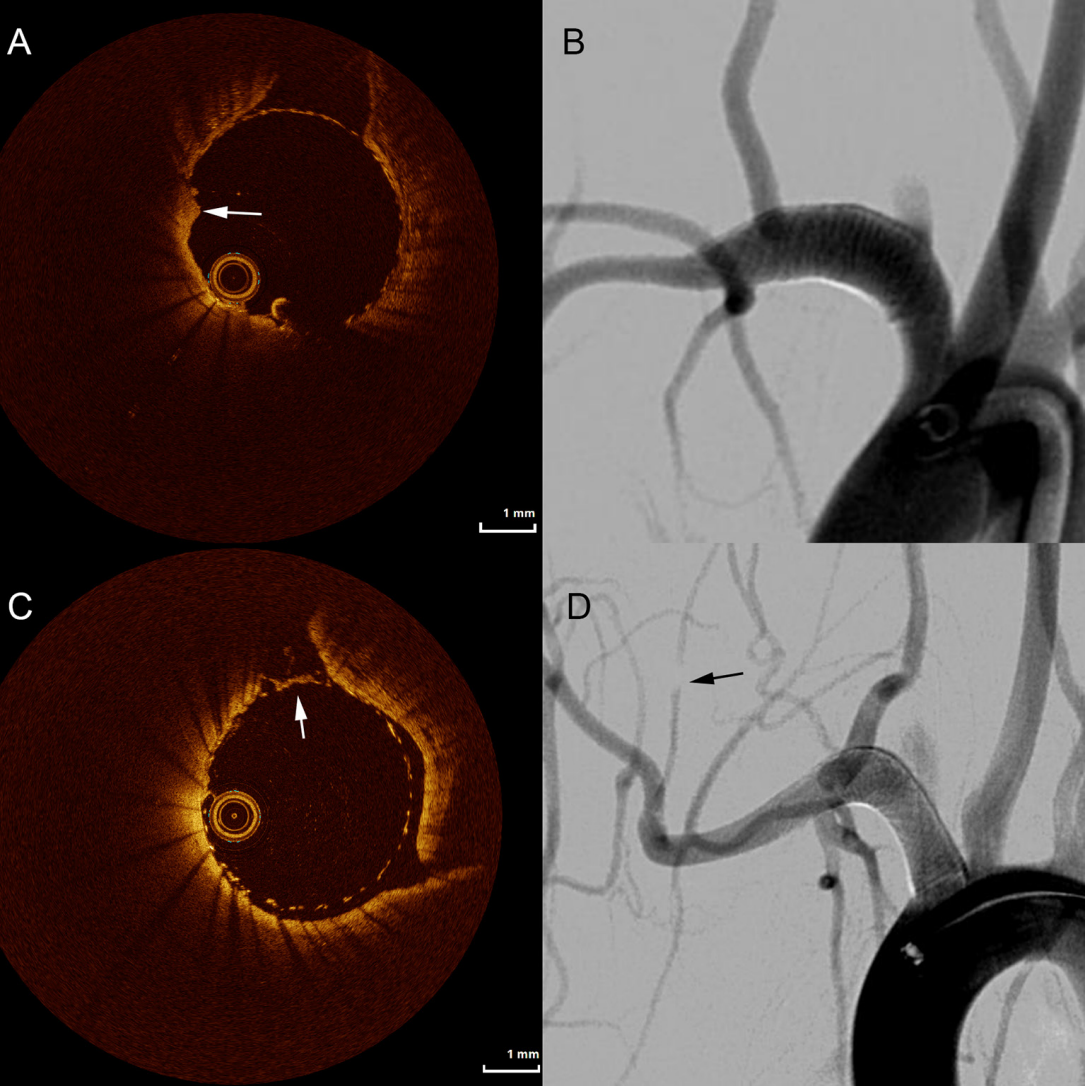
Following the implant and angioplasty of a classic Pipeline embolization device in an animal not receiving dual antiplatelet therapy, significant thrombus accumulation is seen distal to the aneurysm (A, arrow) that is not observed on standard digital subtraction angiography (B). In another animal of the same group, thrombus developed over a covered side branch (C, arrow) without filling deficit on angiography (D). Angiography shows a filling deficit downstream from the device (D, arrow).

This animal study deployed OCT in order to identify small but potentially harmful thrombi on the surface of flow diverters. The identified thrombi size ranged from 0.4 mm to 1.2 mm. Despite DSA failing to reveal clots on the surface of the FD (figure 3), OCT indicated the presence and exact locations of thrombi, potentially serving as guidance for more targeted treatment. It has further been shown that DSA fails to depict FD malapposition in rabbit models compared with histological evaluation.^10^ The usefulness of OCT in detecting acutely formed microthrombi^11^ and stent apposition^12^ has been extensively studied in cardiology, but currently there is no dedicated neurovascular OCT system, representing an opportunity for technology development. Besides the higher spatial resolution of OCT, the ability to assess the inner lumen of the vessel along with the implanted FD in a 360^o^ view provides additional advantages for detailed analysis.^13 14^ In contrast, the two-dimensional view of DSA can lead to false-negative identification of microthrombi. In general, the morphology of the clot identified by OCT was an irregular layer that could be observed in multiple slices without significant luminal narrowing. Importantly, microthrombi were frequently observed at the ostia of small side branches, representing an important potential source for thromboembolic complications.

On the initial OCT, immediately following device implant, there was no difference in clot formation between the cPED and sPED groups and, more interestingly, it appeared that DAPT did not have any effect on preventing clot formation during any of the different stages of the procedure. However, by performing angioplasty after device implant and exposing the thrombogenic subendothelial layer of the vessel, sPED showed significantly fewer newly formed thrombi than cPED, supporting our hypothesis that PC surface modified FDs can be protective against acute clot formation, especially after angioplasty. A more profound result was the statistically significant reduction of microthombi at the FD covered orifice of small branch arteries when sPED was used. Despite a reduction of thrombi along the luminal surface of the device, sPED did not alter the presence of thrombi acutely at the location of the aneurysm or aneurysm occlusion rates on 30-day follow-up angiography.

These data may lead to the conclusion that, in this model, avoiding angioplasty is critical for the prevention of microthrombi along the surface of the device. However, our study design cannot confirm this conclusion since the pre-angioplasty OCT was performed immediately following FD deployment whereas the post-angioplasty delayed imaging by more than 30 min. Although angioplasty was specifically deployed to create an aggressive but clinically relevant pro-thrombotic environment to assess the benefit of the modified FD, time from implant may have also contributed to platelet aggregates. Our study design did not include a no angioplasty group with delayed OCT acquisition, since this would have doubled the number of animals required without contributing significant information to testing the stated hypothesis. Notably, malapposition of FDs is clearly underappreciated by existing imaging systems and critical for aneurysm occlusion following FD placement.^10^ Numerous techniques are used for improving FD apposition such as gentle manipulation with the delivery microcatheter, intermediate distal access catheter,^15^ balloon angioplasty,^15 16^ or the use of self-expanding stents to pin the FD.^17^ We deployed angioplasty since, of these techniques, it offers reproducibility.

Platelet function testing and titration of DAPT for individual patients has been advocated to reduce thromboembolic or bleeding complications.^18–20^ However, in both coronary stenting^21^ and neurointerventional treatment of aneurysms,^22 23^ pre-procedural platelet aggregation testing has failed to demonstrate benefit in clinical outcomes. Due to the variability of patient response to standard DAPT regimens, there is a need for endovascular devices with reduced thrombogenicity that can produce a predictable platelet response. Our study shows that the sPED technology is an important development towards this goal.

Our study has limitations. Although the rabbit model is well established for studying antiplatelet effects of clopidogrel and aspirin,^24–28^ these animals had a standard laboratory diet, were medicated on a precise schedule, and did not have comorbidities. Therefore, the response of the model to DAPT may not translate to clinical populations. Due to the location of the implant in the subclavian artery, we could not correlate thrombus formation observed on OCT with clinical sequelae. Despite low porosity implants in small diameter arteries, we did not observe device thrombosis in the absence of DAPT. Finally, we did not perform acute histology following the implant procedure, as this would not allow assessment of 30-day aneurysm occlusion and patency of parent and branch arteries. Although we cannot confirm with histology the OCT findings, we relied on the extensive literature for appearance of thrombus on endoluminal devices using OCT.^12^
